# Platelet proteome reveals specific proteins associated with platelet activation and the hypercoagulable state in β-thalassmia/HbE patients

**DOI:** 10.1038/s41598-019-42432-2

**Published:** 2019-04-15

**Authors:** Puangpaka Chanpeng, Saovaros Svasti, Kittiphong Paiboonsukwong, Duncan R. Smith, Kamonlak Leecharoenkiat

**Affiliations:** 1 Oxidation in Red Cell Disorders and Health Task Force, Department of Clinical Microscopy, Faculty of Allied Health Sciences, Chulalongkorn University, Bangkok, Thailand; 2 Thalassemia Research Center, Institute of Molecular Biosciences, Mahidol University, Bangkok, Thailand; 3 Molecular Pathology Laboratory, Institute of Molecular Biosciences, Mahidol University, Bangkok, Thailand

## Abstract

A hypercoagulable state leading to a high risk of a thrombotic event is one of the most common complications observed in β-thalassemia/HbE disease, particularly in patients who have undergone a splenectomy. However, the hypercoagulable state, as well as the molecular mechanism of this aspect of the pathogenesis of β-thalassemia/HbE, remains poorly understood. To address this issue, fifteen non-splenectomized β-thalassemia/HbE patients, 8 splenectomized β-thalassemia/HbE patients and 20 healthy volunteers were recruited to this study. Platelet activation and hypercoagulable parameters including levels of CD62P and prothrombin fragment 1 + 2 were analyzed by flow cytometry and ELISA, respectively. A proteomic analysis was conducted to compare the platelet proteome between patients and normal subjects, and the results were validated by western blot analysis. The β-thalassemia/HbE patients showed significantly higher levels of CD62P and prothrombin fragment 1 + 2 than normal subjects. The levels of platelet activation and hypercoagulation found in patients were strongly associated with splenectomy status. The platelet proteome analysis revealed 19 differential spots which were identified to be 19 platelet proteins, which included 10 cytoskeleton proteins, thrombin generation related proteins, and antioxidant enzymes. Our findings highlight markers of coagulation activation and molecular pathways known to be associated with the pathogenesis of platelet activation, the hypercoagulable state, and consequently with the thrombosis observed in β-thalassemia/HbE patients.

## Introduction

β thalassemia/HbE disease is the most common form of severe β thalassemia, responsible for approximately one-half of all severe β-thalassemia cases worldwide. The disease results from the co-inheritance of a β-thalassemia allele and hemoglobin E, the most prevalent structural β-globin variant in Southeast Asia. Patients that carry β-thalassemia/HbE show a clinical severity ranging from a severe transfusion-dependent thalassemia major to thalassemia intermediate^1^. The pathophysiology of β-thalassemia/HbE is mainly related to the accumulation of excess α-globin chains and its degradation products such as heme or hemin due to the imbalance of globin chain production. Ineffective erythropoiesis, peripheral hemolysis and iron overload are important factors responsible for the clinical manifestation of this disease^2,3^.

Recently an increased risk for developing hypercoagulopathy, resulting in thrombotic events, has been observed in β-thalassemia/HbE patients^4^. The thrombosis mainly occurs in the venous system and manifests as a stroke, deep vein thrombosis or pulmonary embolism^5^. The hypercoagulopathy in patients with thalassemia has been attributed to several risk factors including abnormal RBCs, chronically activated platelets, enhanced platelet aggregation, iron overload, splenectomy, and increased prothrombin generation^6–9^. Platelets normally circulate in a non-activated state and play an important role in homeostasis and thrombin generation. The mechanism by which activated platelets induce thrombosis in β-thalassemia/HbE disease has not been fully elucidated. We sought to determine the platelet proteome related to the underlying mechanism of platelet activation and the hypercoagulable state in the β-thalassemia/HbE disease by analyzing peripheral blood platelets isolated from healthy volunteers as compared to peripheral blood platelets from β-thalassemia/HbE patients with and without splenectomy. Our study provides novel insights to the underlying mechanism and pathophysiology of this disease.

## Results

### Clinical characteristic of β-thalassemia/HbE patients and healthy volunteers

Twenty-three β-thalassemia/HbE patients (15 non-splenectomized and 8 splenectomized patients) and 20 healthy volunteers were enrolled in this study. The hematologic data of all subjects are summarized in Table 1. The patients groups had lower Hb, Hct and mean corpuscular volume (MCV), and marked increases in the numbers of white blood cells, platelets, nucleated RBCs, and reticulocytes as compared to normal subjects. Comparison of hematologic parameters between β-thalassemia/HbE patients with and without splenectomy was also undertaken. As compared to the non- splenectomized group, the splenectomized patients showed lower Hb, Hct and MCV, but higher RDW and marked increases in the numbers of WBC, reticulocyte, nRBCs and platelets.

**Table 1:** Comparison of lab finding between healthy volunteers and β-thalassemia/HbE Patients.

Laboratory parameters	Healthy volunteers (n = 20)	β-thal/HbE patients (n = 23)	*p-value*	Non-splenectomy (n = 15)	Splenectomy (n = 8)	*p-value*
Age (mean ± SD)	28.0 ± 6.1	35.33 ± 9.3	0.3224	28.7 ± 6.2	34.0 ± 8.8	0.8750
Male female (n (%))	9 (45.0) 11 (55.0)	12 (52.2) 11 (47.8)	0.7626	7 (46.7) 8 (53.3)	5 (62.5) 3 (37.5)	0.6668
RBC × 10^6^ cell/ul	4.3 ± 0.8	4.1 ± 1.0	0.3300	5 ± 1.0	3 ± 0.4	0.0036
Hb g/dL	14.0 ± 1.1	7.7 ± 1.2*	<0.0001	8.3 ± 1.1	6.5 ± 0.6	0.0005
Hct %	41.9 ± 2.4	26.2 ± 3.7*	<0.0001	27.6 ± 3.4	23.4 ± 2.2	0.0059
MCV fL	88.4 ± 4.2	66.4 ± 9.3*	<0.0001	62.9 ± 9.3	72.7 ± 5.3**	0.0138
RDW %	12.3 ± 0.4	22.1 ± 2.3*	<0.0001	22.2 ± 2.2	24.8 ± 1.1**	0.0067
NRBCs	0	133.0 ± 64.9*	<0.0001	4.5 ± 1.9	364.2 ± 295.4**	<0.0001
Reticulocyte count %	0.9 ± 0.1	5.4 ± 4.5*	<0.0001	2.0 ± 0.6	11.1 ± 1.0**	<0.0001
WBC Count × 10^3^ cell/µL	5.9 ± 0.9	8.7 ± 4.3*	0.0072	6.1 ± 1.9	13.7 ± 2.7**	<0.0001
Platelet count × 10^3^ cell/uL	289 ± 49	343.7 ± 231.2	0.3075	191 ± 65	630 ± 126**	<0.0001
Serum ferritin (ng/uL)	76.2 ± 37.8	979.1 ± 701.6*	<0.0001	512.2 ± 319.6	1757 ± 382.8**	<0.0001

Abbrevation: RBC, red blood cell; Hb, hemoglobin; Hct, hematocrit; MCV, mean corpuscular volume; RDW, Red blood cell distribution; WBC count, white blood cell count. Values represent mean ± SD. ^*^Significant difference compared between healthy volunteers and β-thalassaemia/HbE patients at *p* < 0.05. ^**^Significant difference compared between non-splenectomy and splenectomy β-thalassaemia/HbE patients at *p* < 0.05.

### Increased platelet activation in β-thalassemia/HbE patients

**Figure 1:**
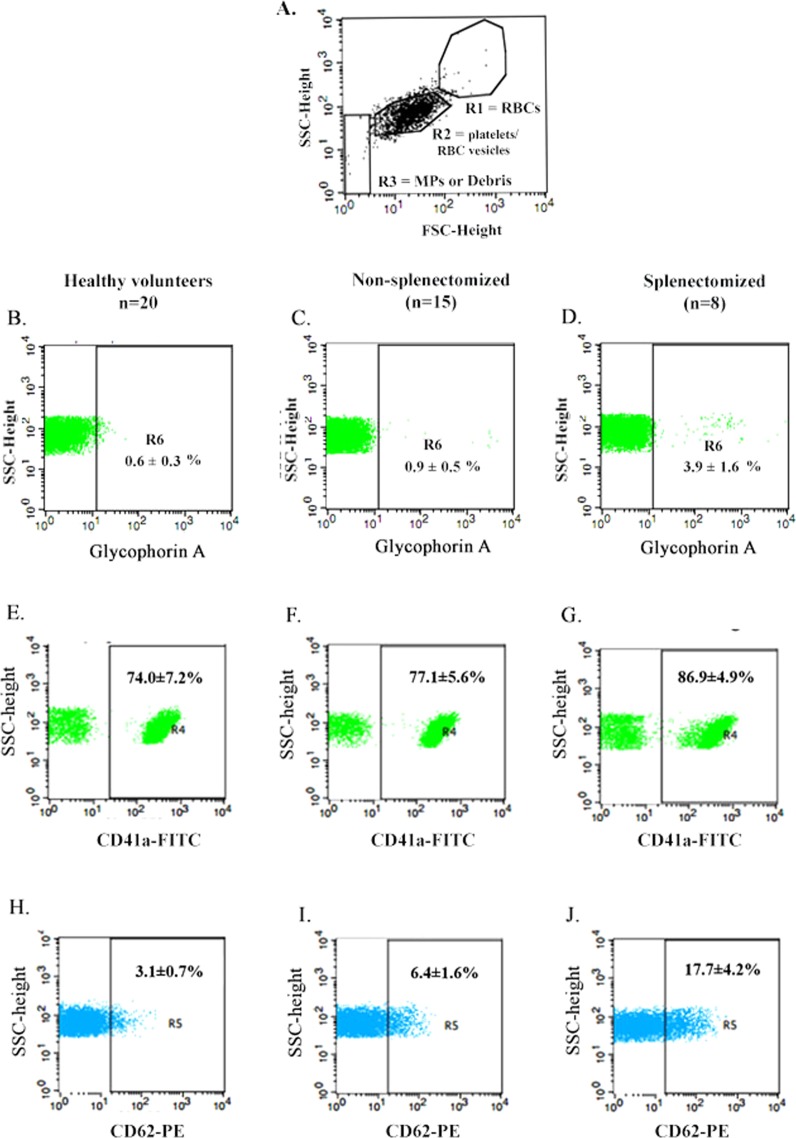
Purity of isolated platelets and level of platelet activation in β-thalassemia/HbE patients and healthy volunteers. After completing the isolation process, the isolated platelet fractions were analyzed by flow cytometry. Plotting between FSC and SSC displays the population of isolated platelets in the R2 region, while the R1 region is the population of red blood cells, and the R3 region is the fraction of microparticles or debris (**A**). The percentages of contaminating RBC vesicles in each subject group were analyzed by plotting between SSC and glycophorin A positives, which were gated from cells at the R2 region (**B**–**D**). The percentages of platelets markers were gated from R2 into R4 region (**E**–**G**). The levels of platelet activation for each subject groups were represented by the percentages of CD62P positive in the R5 region (**H**–**J**).

By using flow cytometry, the isolated platelet populations were analyzed using plots of forward scatter (FSC-H) and side scatter (SSC-H). The population of isolated platelets in each subject group is found in the R2 region of the scatter plot (Fig. 1A). The platelet population in the R2 region was gated to analyze the percentage of contaminating RBC vesicles which are represented by the percentage of glycophorin A positive entities. The result showed that the platelet fraction of all subject groups contained contaminating RBC vesicles of less than 5%, including 0.6 ± 0.3% in samples from normal controls, 0.9 ± 0.5% in samples from non-splenectomized β-thalassemia/HbE patients and 3.9 ± 1.6% in samples from splenectomized β-thalassemia/HbE patients (Fig. 1B–D). These results indicate that the purity of isolated platelets is high, and they could be used for the other experiments. Entities at the R2 region were further gated and analyzed for the expression of a platelet specific marker (CD41a+) (Fig. 1E–G). The percentage of activated platelets (CD62P+) was gated from the R4 region (Fig. 1H–J). Samples from β-thalassemia/HbE patients showed a significantly higher percentage of activated platelets (CD62P+) as compared to samples from healthy volunteers and the samples from splenectomized patients showed a higher level than the samples from non-splenectomized patients.

### Levels of prothrombin fragment 1 + 2 and its correlation with the platelet activation

**Figure 2:**
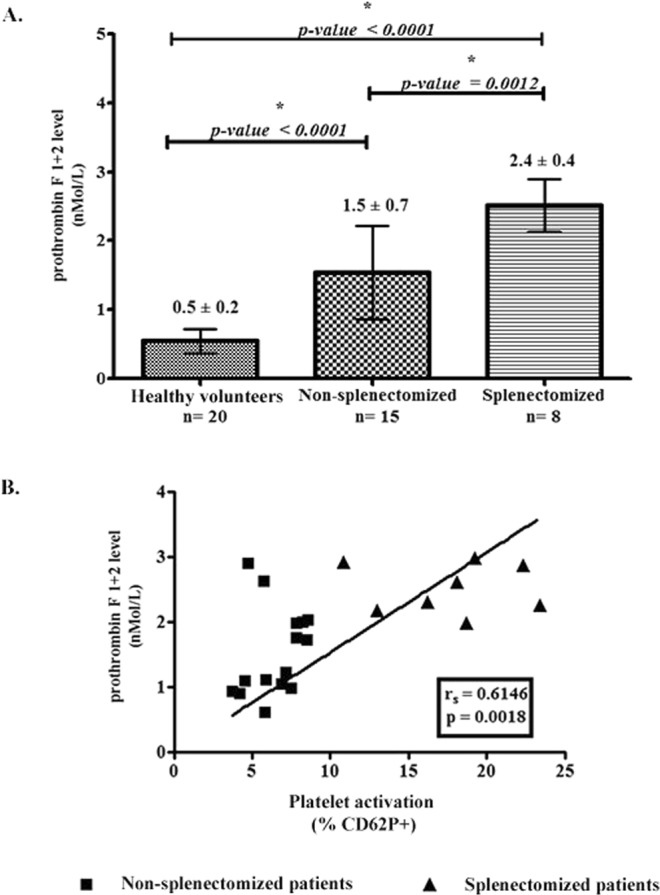
Level of prothrombin fragment 1 + 2 and it’s the correlation with the platelet activation. The prothrombin fragment 1 + 2 levels of 8 splenectomized patients, 20 healthy volunteers and 15 non-splenectomized patients were detected by ELISA. The results of healthy volunteers, β-thalassemia/HbE patients and the reference value are shown in the columns with dashed lines represent mean ± SD (**A**). The spearman correlation coefficient was calculated between percentage of platelet activation (CD62P-positive) and levels of prothrombin fragment 1 + 2 (**B**).

The hypercoagulable state was evaluated by measuring the level of prothrombin fragment 1 + 2. Non-splenectomized β-thalassemia/HbE patients had threefold higher prothrombin fragment 1 + 2 levels than healthy volunteers. The highest levels of prothrombin fragment 1 + 2 were found in the splenectomized β-thalassemia/HbE patients (Fig. 2A), and the levels of prothrombin fragment 1 + 2 in the splenectomized patients were significantly higher than the levels found in the non-splenectomized patients. The correlation between levels of platelet activation and prothrombin fragment 1 + 2 among β-thalassemia/HbE patients was analyzed (Fig. 2B) and it was seen that the levels of platelet activation were closely correlated with the levels of prothrombin fragment 1 + 2 (r = 0.6146, p = 0.0018).

### Comparative differential platelet proteome between healthy volunteers and β-thalassemia/HbE patients

**Figure 3:**
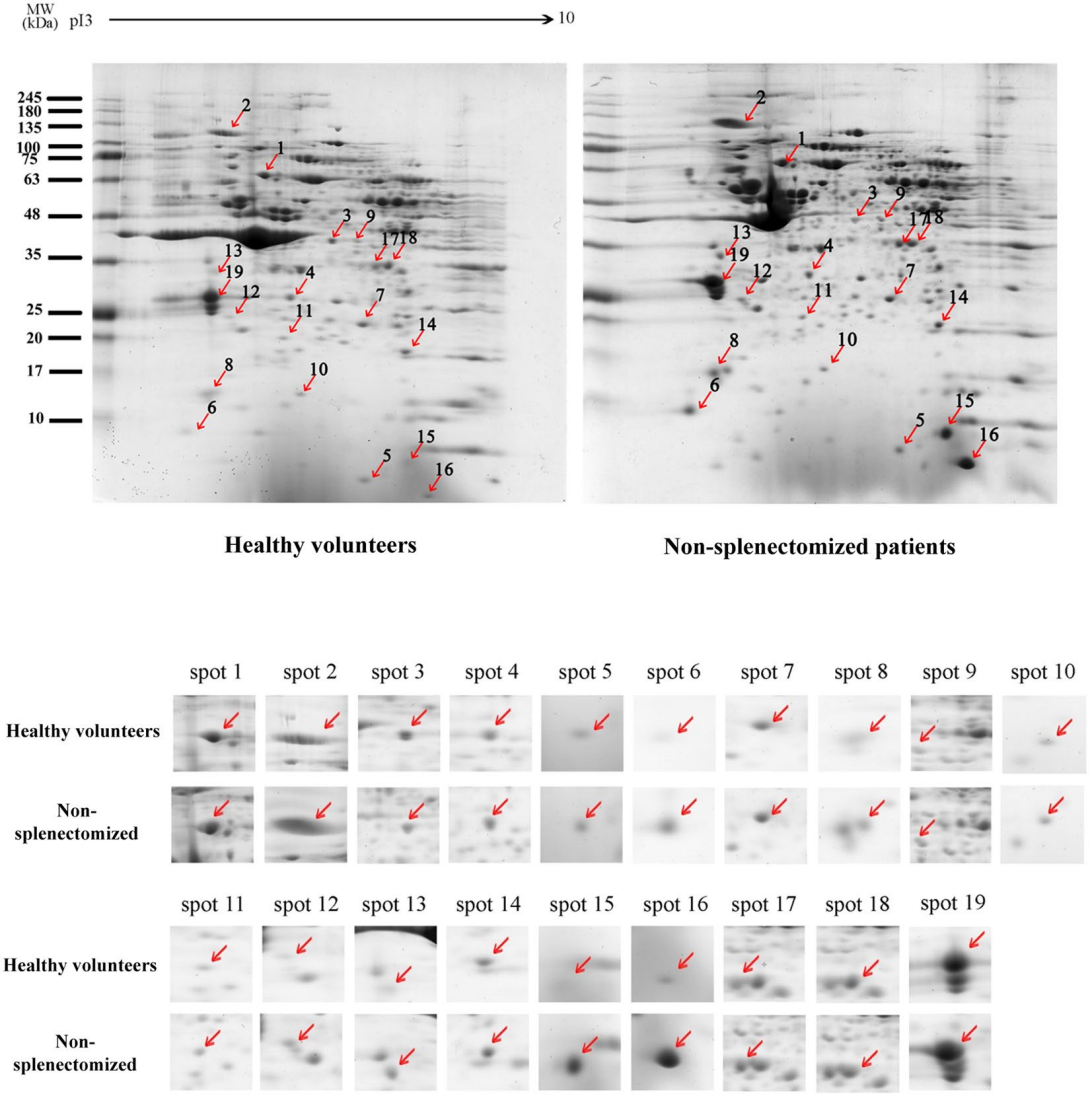
Platelet proteomes of non-splenectomized β-thalassemia/HbE patients and healthy volunteers. Platelet proteins compared between 4 non-splenectomized β- thalassemia/HbE patients and 4 healthy volunteers were subjected to 2D-electrophoresis following which gels were stained with Brilliant Blue Coomassie G250. The comparative 2D gels are representative of 4 replicates for each subject group. The resultant proteins spots were detected and analyzed by Image Master 2D-Platinum v7 software (Amersham Biosciences). The red arrows show 19 differential spots (p < 0.05) when comparing between the 2 sample groups. Eighteen of the protein spots were up-regulated and one protein spot was down-regulated (spot no. 3). The 19 protein spots that changed significantly were cropped from the same gel and enlarged.

The platelet proteome of 4 non-splenectomized β-thalassemia/HbE and 4 healthy volunteers were analyzed by 2D gel electrophoresis (Supplementary information). The spot expression levels were compared by analyzing the intensity, volume, and area of the protein spots. A total of 19 differentially regulated protein spots were identified, of which 18 spots were up-regulated and 1 spot was down-regulated in non-splenectomized β-thalassemia/HbE as compared to normal controls (Fig. 3). The spots were excised from the gels and subjected to tryptic in-gel digestion followed by mass spectroscopic analysis of the resultant peptides. A total of 19 proteins were identified as shown in Table 2. The down-regulated protein was identified as leukocyte elastase inhibitor. The largest groups of up-regulated proteins were cytoskeleton membrane proteins (F-actin -capping protein subunit beta, myosin light polypeptide 6, myosin regulatory light chain 12A, myosin-9, Rho GDP-dissociation inhibitor 2, transgelin-2, PDZ and LIM domain protein 1, and tropomyosin alpha-4 chain). The others upregulated proteins were a chaperone protein (heat shock protein 70 kDa protein), a fibrinogen receptor (integrin alpha IIb), an immune cell activation marker (beta-2-microglobulin), a globin protein (hemoglobin subunit beta) and platelet factor 4 (or chemokine (C-X-C motif) ligand 4 (CXCL4)).

**Table 2:** Differential proteins compared between non-splenectomized β- thalassemia/HbE patients and healthy volunteers.

No	Protein name	MW(Da)	PI	Score	%Coverage	Fold change β/HbE vs healthy volunteers	Function
1	Heat Shock 70 kDa protein	70294	5.48	1033	25	1.41	-Chaperone protein
2	Integrin Alpha-IIb	114446	5.21	853	11	1.36	-Fibrinogen Receptor -Platelet activation and aggregation
3	Leukocyte Elastase Inhibitor	42829	5.9	2557	44	−1.11	-Serine protease inhibitor
4	F-actin -capping protein subunit beta	31616	5.36	2034	39	1.16	-Cytoskeleton
5	Beta-2-Microglobulin	13820	6.06	42	16	1.12	-Immune activation
6	Myosin light polypeptide 6	17090	4.56	397	21	1.45	-Cytoskeleton
7	Peroxiredoxin 6	25133	6	2187	76	1.05	-Antioxidant enzyme
8	Myosin regulatory light chain 12 A	19839	4.67	527	41	1.06	-Cytoskeleton
	Myosin regulatory light polypeptide 9	19871	4.8	503	23	1.06	-Cytoskeleton
9	Biliverdin reductase A	33692	6.06	203	26	1.09	-Heme synthesis
10	Actin -related protein 2/3 complex sub unit 5	16367	5.47	1147	47	1.32	-Cytoskeleton
11	Glutathione S-transferase P	23569	5.43	732	18	1.18	-Antioxidant enzyme
12	Rho GDP-dissociation inhibitor 2	23031	5.1	1229	30	1.22	-Cytoskeleton
13	Tropomyosin alpha- 1 chain	32746	4.69	134	13	1.04	-Cytoskeleton
14	Transgelin- 2	22548	8.41	3391	64	1.40	-Cytoskeleton
15	Hemoglobin subunit beta	16102	6.75	985	58	3.90	-Globin
16	Platelet factor 4 (CXCL4 or PF4)	14171	9.04	792	45	5.42	-Chemokine -Platelet activation -Heparin binding protein
17	PDZ and LIM domain protein 1	36505	6.56	2118	48	1.02	-Cytoskeleton
18	PDZ and LIM domain protein 1	36505	6.56	2590	44	1.05	-Cytoskeleton
19	Tropomyosin alpha- 4 chain	28619	4.67	889	57	1.09	-Cytoskeleton

### Western blot analysis of integrin αIIb and platelets factor4

**Figure 4:**
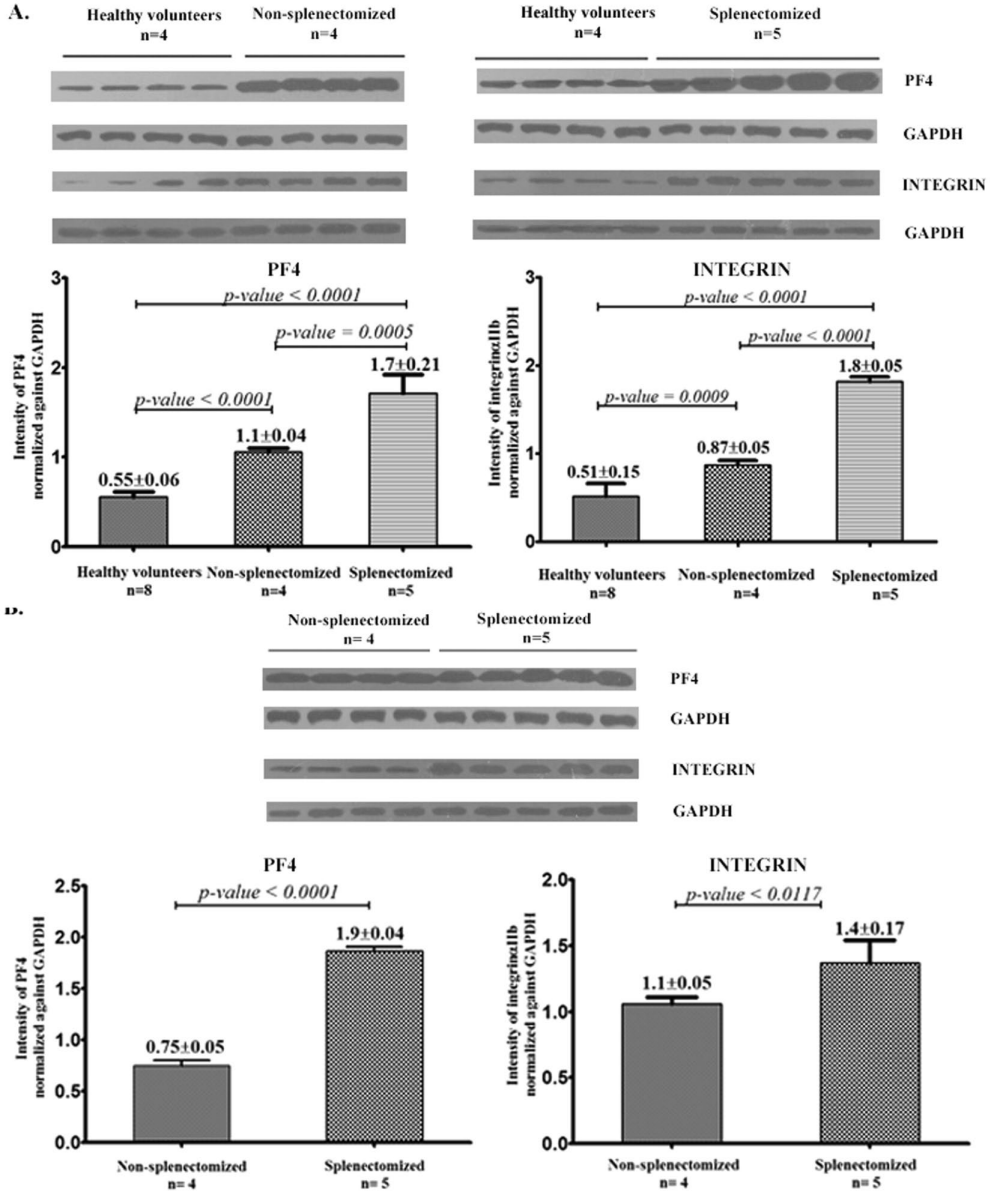
Expressions of PF4, Integrin αIIb and GAPDH detected by western blot analysis. Representative western blot analysis of PF4 and Integrin αIIb, normalized against GAPDH in healthy volunteers compared to the β- thalassemia/HbE patients are shown in figure (**A**). The comparison of protein expression between non-splenectomized β-thalassemia/HbE patients and splenectomized β- thalassemia/HbE patients are shown in figure (**B**). Band intensities for PF4 and Integrin αIIb were qualified and normalized against GAPDH and are shown as mean ± SD. The WB analysis for each protein compared between each subject group (healthy volunteer vs non-splenectomized patients, normal vs splenectomized patients and non-splenectomized vs splenectomized patients) was run in the same gel and transferred to the same nitrocellulose membrane. All membranes of the same comparing protein were exposed in the same X-ray film.

To validate the 2D proteomic analysis results, the levels of two differentially expressed proteins (platelet factor 4 and integrin αIIb) were measured by western blot analysis (Fig. 4). Platelet proteins were isolated from independent subjects (i.e. subjects whose samples were not used for the 2D analysis), including four non-splenectomized β-thalassemia/HbE patients, five splenectomized β-thalassemia/HbE patients and 8 healthy volunteers. The membranes were probed with antibodies directed to integrin αIIb and platelet factor 4 and normalized against GAPDH (Supplementary information). The results were consistent with 2D gel analysis in that the levels of integrin αIIb and platelet factor 4 were significantly increased in the non-splenectomized β-thalassemia/HbE patients when compared to healthy volunteers. Moreover, the splenectomized β-thalassemia/HbE had a higher level of those proteins as compared to the non-splenectomized patients.

## Discussion

Hypercoagulability leading to a thromboembolic event is a significant factor influencing the morbidity and mortality of β-thalassemia patients. Several studies have shown higher platelet activation levels in β-thalassemia intermedia and splenectomized thalassemia patients than in healthy volunteers^8,10–14^, and previous studies have indicated that the CD62P (P selectin) is a reliable marker for measuring platelet activation^15^. Confirming a previous report^16^, we found significantly elevated platelet activation (CD41+/CD62P+) in non-splenectomized and splenectomized β-thalassemia/HbE patients. Increased levels of prothrombin fragment 1 + 2 (F1 + 2), which is an indicator of the hypercoagulable state, were also detected at higher levels in β-thalassemia/HbE patients as compared to healthy volunteers. While thrombosis is a known post-splenectomy consequence, our findings indicate a significant increase in platelet activation, hypercoagulation, serum ferritin as well as the up-regulation of platelet proteins in non-splenectomized patients, suggesting a closer monitoring of thrombosis in this group of patients is also needed. Moreover, it is noted that the expression of CD62P mediates the binding, rolling, and weak adhesion of platelets to leukocytes via P-selectin glycoprotein ligand 1 (PSGL-1)^17^. Previous study found that the levels of platelet-monocyte and platelet-neutrophil aggregation were elevated in β-thalassemia/HbE patients who had a hypercoagulation state. This result suggests that the platelet-leukocyte interaction might contribute to thrombin generation leading to the hypercoagulable state in β-thalassemia^18^.

In order to identify platelet proteins associated with platelet activation or the hypercoagulable state in the β-thalassemia/HbE disease, a proteomic analysis was undertaken. The proteomic analysis showed 19 differentially expressed spots, which were identified by mass spectrometry as 19 different proteins. The proteomic analysis was not undertaken on platelets from splenectomized β-thalassemia/HbE patients as despite repeated attempts we were unable to obtain consistent isoelectric focusing, possibly as a result of high levels of coagulation factors in these samples. However, the proteins identified as differentially expressed between non-splenectomized β-thalassemia/HbE patients and healthy volunteers included a number of cytoskeletal proteins (F-actin -capping protein subunit beta, myosin light polypeptide 6, myosin regulatory light chain 12A, myosin-9, Rho GDP-dissociation inhibitor 2, transgelin-2, PDZ and LIM domain protein 1, and tropomyosin alpha-4 chain) as well as heat shock protein 70 kDa protein, integrin alpha IIb, beta-2-microglobulin, hemoglobin subunit beta, platelet factor 4 (or chemokine (C-X-C motif) and leukocyte elastase inhibitor. Of these proteins, all were shown to be up-regulated in β-thalassemia/HbE patients with the exception of leukocyte elastase inhibitor which was down-regulated.

One previous study has investigated the differential platelet proteome in the β-thalassemia/HbE disease^19^. That study identified 5 proteins (Hsp70, protein, disulfide-isomerase, eukaryotic translation initiation factor 5A-1, peroxiredoxin-2 and superoxide dismutase [Cu-Zn]). This study, and the study by Karmakar^19^, only identified one protein in common, namely Hsp70. However, as reviewed elsewhere multiple studies determining the differences in proteomes between β-thalassemia/HbE patients and healthy volunteers rarely identify common proteins as a consequence of the usage of different samples sources (erythrocytes, platelet free plasma derived microparticles, plasma, etc) and different analysis methodologies, as extensively reviewed elsewhere^20^. In this regard we found higher serum ferritin levels in splenectomized patients than in non-splenectomized patients and healthy volunteers in contrast to the previous study^19^. Similarly, while the previous study^19^ showed higher levels of protein C, which is a prothrombinase complex inhibitor, in splenectomized patients as compared to healthy volunteers and non splenctomized patients, our study showed increase activation of the prothrombinase complex as shown by the increased levels of prothrombin fragment 1 + 2 in the splenectomized patients.

As noted above, the majority of the differentially expressed proteins (10 out of 19) detected in this study were membrane skeleton proteins such as actin, myosin, tropomyosin and trangelin. These proteins are highly abundant in platelets and regulate contractile properties, indicating cytoskeleton re-organization during platelet activation^21^. In addition to the up-regulation of cytoskeletal proteins in platelets from β-thalassemia/HbE patients, we observed the up-regulation of integrin, a transmembrane receptor protein that acts as a bridge between the extracellular matrix and the cytoskeletal membrane that mediates signal transduction in activated platelets, resulting in platelet shape changes and platelet aggregation^22^. The up-regulation of integrin proteins is mediated by ADP, thromboxane A2 though a G protein –mediated signaling pathway which includes Rho GDP-dissociation inhibitor 2, which also seen as up-regulated in this study. This pathway activates a conformational change in the extracellular domains of proteins such as fibrinogen or von Willebrand factor, and fibrinogens act as bridges between platelets to generate platelet aggregation^23^. Additionally, as noted above we also found the up-regulation of Hsp70, a specific chaperone that forms complexes with other co-chaperones in maintaining hemostasis and is associated with intracellular organization of signaling systems and platelet function^24^, and expression of Hsp70 is an endogenous mechanism by which living cells adapt to stress^25^. Moreover, Hsp70 is associated with the inside-out activation of integrin-αIIbβ3 and the activation of platelet aggregation as well as granule secretion and platelet formation under conditions of physiological shear^26^. The consequences of signal transduction related to platelet integrin-αIIb and glycoprotein membrane changes are release of its granules including ADP (dense granules) and P-selectin and platelet factor 4 (PF4) (alpha-granules)^27^ which were also up-regulated in this study.

Interestingly, the expression of platelet factor 4 (PF4) was a significantly higher in non-splenectomized and splenectomized patients. PF4 is a cytokine that is specifically released from alpha-granules of activated platelets^27^. PF4 plays a role in inflammation, atherosclerosis and thrombosis by neutralizing heparin anticoagulation to increased clot formation stability^28–30^ and heparin-induced thrombocytopenia (HIT)^31^. The up-regulation of PF4 protein is a consequence of cytoskeleton, Hsp70 and integrin-αIIb protein changes, and the proteomic and western blot analyses showed very clearly increased levels in the β thalassemia/HbE patients. This result indicates that PF4 could be used as a potential diagnostic or predictive marker for the presence of thrombosis in β thalassemia/HbE patients. The others proteins identified as differentially expressed were related to heme synthesis (Biliverdin reductase A) and antioxidant enzymes (Peroxiredoxin 6, Glutathione S-transferase P) which may reflect an increase of free radical levels and consequent induction of antioxidant enzymes to protect against apoptosis^32^.

**Figure 5:**
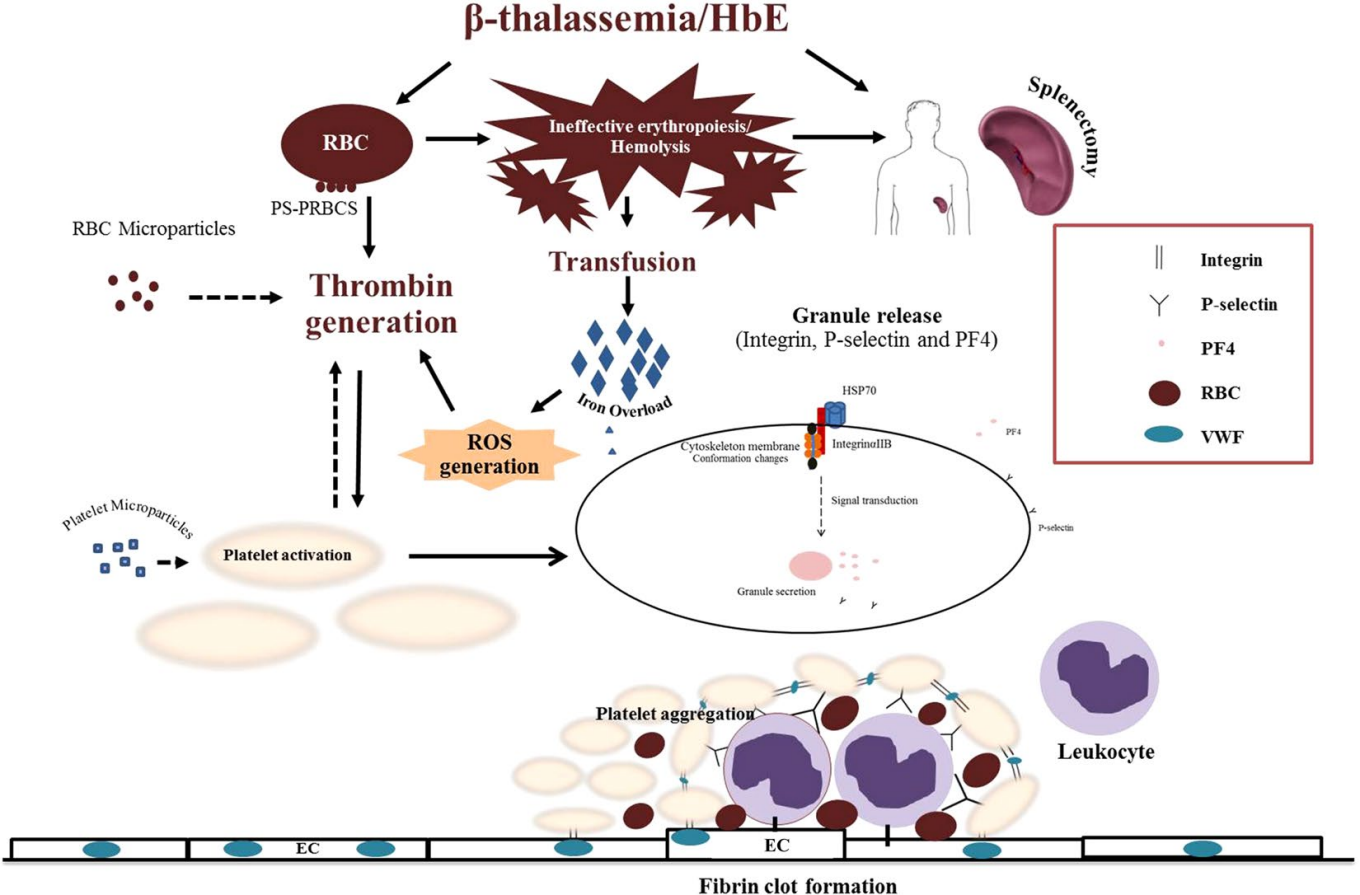
Schematic model illustrating a possible mechanism of hypercoagulable state in β-thalassemia/HbE. We proposed that the pathophysiology of β-thalassemia/HbE such as abnormal RBC and ineffective erythropoiesis induced PS- RBCs, hemolysis and splenectomy status in patients result in iron accumulation and increased ROS. These factors can indirectly and directly activate platelet alterations including platelet shape changes, secretion and aggregation leading to hypercoagulabilty in β-thalassemia/HbE patients.

Overall, our study confirmed the association of platelet activation and hypercoagulation, and identified a specific marker potentially predicting thrombosis which could improve early diagnosis and treatment intervention in β-thalassemia/HbE patients. We found significantly increased platelet activation, prothrombin fragment 1 + 2 levels and serum ferritin levels. Previous studies have shown a correlation of prothrombin fragment 1 + 2 levels and platelet activation with phosphatidylserine exposure on RBC^33,34^. Thus, platelet activation may be associated with several factors, including abnormal RBCs, RBC exposed phophotidylserine, micropartcles, hemolysis, iron overload and ROS resulting in chronic platelet activation and hypercoagulability in β-thalassemia/HbE patients (Fig. 5). It has been proposed that RBC hemolysis products (heme, hemin and iron) induce the generation of reactive oxygen species (ROS) such as superoxide radicals and hydrogen peroxide^35^ and that ROS are believed to affect platelet function and promote platelet activation both directly through the action of superoxide radicals, and indirectly through nitric oxide inhibition which contributes to platelet aggregation and thrombus formation^36^. Additionally, abnormal RBCs membranes lose phospholipid asymmetry, and expose phosphatydyl serine (PS) both on their membranes and on RBC derived microparticles which are shed into the blood circulation^37^. RBCs derived microparticles, a procoagulant, can enhance prothrombin complex formation leading to platelet activation which can additionally produced platelet derived microparticles that activate platelets^8,38,39^. When platelets are activated, the platelet skeleton membranes changes and they attach to the extracellular matrix and secrete chemokines and aggregate to others cells such leukocyte and endothelial cells leading to the formation of a plug^40^. Our results have shown the alteration of many platelet proteins leading to an understanding of pathways contributing to the hypercoagulable state in β-thalassemia/HbE patients. We suggest that possible preventive approaches for β-thalassemia/HbE patients with a hypercoagulable state or thrombosis include decreasing the level of RBCs exposing phosphatidylserine RBC, reducing ROS using antioxidants and inhibiting the coagulation pathway using anticoagulants.

## Materials and Methods

### Blood samples collection

This study subjects consisted of 23 β-thalassemia/HbE patients and 20 healthy volunteers. There was no significant difference in gender and age distribution between patients and healthy volunteers. Approval for the study was obtained from the Ethics Review Committee for Research Involving Human Research Subjects, Health Science Group, Chulalongkorn University with the certificate of approval no. 196/2016. Signed informed consent was obtained from all participants before blood collection. All methods were performed in accordance with the relevant guidelines and regulation of Chulalongkorn University. Complete blood cell counts were undertaken using a Sysmex XE 5000 hematology analyzer (Sysmex Corporation, Kobe, Japan). Diagnosis of β thalassemia/HbE was done by automated high performance liquid chromatography (HPLC) hemoglobin typing (VARIANT^TM^, Biorad, Hercules. CA, USA) and reverse dot blot hybridization technique with the allele-specific oligonucleotide (ASO) probes followed standard published protocols^41^. Healthy volunteers were screened to be normal by complete blood cell count and hemoglobin analysis. The patients had not received a blood transfusion for one month, and had stopped medication for at least two weeks prior to blood sample collections. Serum ferritin levels were determined using a Ferritin Elisa kit (DiaMetra srl Unipersonel, Boldon, UK).

### Platelet isolation method

Ten microliters of a citrate dextrose acid (ACD) blood samples were centrifuged twice at 200 g for 10 min at room temperature to collect platelet rich plasma (PRP). The platelets in the PRP fraction were inhibited by addition of 2 µM prostagladin E1 (Clayman Chemical, Michigan, USA) and incubation for 10 min at room temperature. After incubation the PRP fraction was centrifuged at 100 g for 10 min at room temperature to depleted red blood cell contamination. After centrifugation, the supernatants were collected in a new tube and centrifuged at 1500 g for 15 min to pellet the platelets. The platelet pellets were washed 3 times and re-suspended in 1 mL of 1x phosphate buffer saline (PBS) buffer, pH 7.4. One hundred microliters of platelet suspensions were kept to analyze platelet activation by flow cytometry. The remaining 900 µL of platelet suspensions were centrifuged at 13,000 rpm, 4 °C for 10 min to pellet the platelets which were kept for proteomic analysis. Levels of RBC vesicle contamination in the purified platelet fraction were measured by determining the levels of glycophorin A (GPA) positive fragments using an anti-human GPA antibody (BD Bioscience, Pharmagen, San Diago, CA) and analysis by flow cytometry.

### Platelet activation analysis by Flow cytometry

After inhibition of *in vitro* platelet activation with prostagladin E1, 5 μL of the samples were stained with 1 µl of a fluorescein isothiocyanate (FITC) conjugated monoclonal antibody against GPIIbIIIa (CD41a, BD Biosciences CA), 1 µl of a phycoerythrin (PE)-conjugated monoclonal antibody against P-selectin (CD62P, BD Biosciences, CA) and 1 µl of a allophycocyanin (APC)-conjugated monoclonal antibody against glycophorin A (GPA, Dakopatts Glostrup, Denmark). The mixtures were incubated for 15 min at room temperature. 150 µL of 1xPBS, pH 7.4 was added to the stained platelets which were immediately analyzed by flow cytometry (FACsClibur, BD Biosciences, San Jose, CA) as described elsewhere^42^.

### Measurement of prothrombin fragment 1 + 2 by ELISA

The presence of a hypercoagulation state was evaluated by measuring the level of prothrombin fragment 1 + 2 using a Human Prothrombin Fragment 1 + 2 (F1 + 2) ELISA Kit (MyBioSource, Inc., San Diego, CA, USA), according to the manufacturer protocol. The color intensity of the reaction was measured at density wavelength of 450 nm by an Infinite Pro M200 ELISA reader (TECAN, Switzerland). All the standard and test sample measurements were undertaken in duplicate. The level of prothrombin fragment 1 + 2 calculated from the standard curve using the Curve Expert software 1.4.

### Two dimension electrophoresis (2D-PAGE)

Platelet fractions were lysed with 150 µL of protein lysis buffer containing 7 M urea, 2 M thiourea, 4% CHAPS, 100 mM DTT, and 1% human protease inhibitor cocktail. A total of 250 µg of soluble proteins containing 2% IPG buffer and 0.5% bromophenol blue were separated in the first dimension using an Immobiline dry strip, pH 3–10 NL, 7 cm. The protein solutions were rehydrated for 14 hours in reservoir slots of the re-swelling tray and isoelectric focusing was performed in a horizontal apparatus Ettan IPGphore3 (GE healthcare Life Sciences, UK). The IPG strips were equilibrated in equilibration buffer containing 100 mM DTT for 30 min and alkylated with 125 mg iodoacetamide (IAA) for 45 min. After equilibration, the proteins were separated in the second dimension using 12.5% polyacrylamide gels using an MiniVE vertical electrophoresis system (GE Healthcare Life Sciences, UK). The 2D gels were stained with 0.1% Coomassie Brilliant Blue G250 in 40% methanol for 24 hours and destained with deionized water for 6 hours to visualize the protein spots. After completion of the staining process, the gels were scanned under visible light at 600 µm/pixel resolution by an Image scanner III (GE Healthcare Life Sciences, UK). Spots were analyzed using Image master V.7 software (GE Healthcare Life Sciences, UK). Statistical analysis was performed by student *t*-test with a *p* value of <0.05 being considered significant.

### Tryptic in-gel digestion and protein identification by LC/MS/MS

Differentially expressed protein spots were picked from the SDS-PAGE gels. The gel pieces were destained in a solution containing 50% ACN in 100 mL of 25 mM ammonium bicarbonate ((NH_4_)HCO_3_) and digested with trypsin for overnight at 37 °C in 25 mM ((NH_4_)HCO_3_). Peptides were extracted in 50 μL of 5% formic acid/50% ACN and then tubes were put into an ultrasonic bath for 15 min and samples subsequently were dried in a speed-vac. Peptide samples were dissolved in 98% H_2_O, 2% ACN and 0.1% formic acid. The digested proteins were analyzed using an LC/MS/MS system consisting of a liquid chromatography part (Dionex Ultimate 3000, Thermo Scientific) in combination with an electro spray ionization (ESI)-ion trap mass spectrometer (amaZon SL, Bruker, Germany). The mass fingerprints were generated and searched against the SwissProt database protein (European Bioinformatics Institute, Cambridge, UK) database using the MASCOT search engine (Matrix Science, London, UK).

### Western Blot analysis

A total of 30 µg of platelet proteins were separated by electrophoresis through 7% SDS-PAGE gels for the detection of integrin α2b protein and 12% SDS-PAGE gels for PF4 protein. After separation, the platelet proteins were transferred and blotted onto 0.2 µm nitrocellulose membranes (GE healthcare Life sciences, UK) using a Semi-Dry Blotter (Mini System, Cleaver Scientific, United Kingdom) and subsequently blocked with 5% non-fat milk in 1xTBS with 0.05% Tween (TBST) for 1 hour at room temperature. For analysis of the expression of PF4 the membranes were incubated with a 1:5000 dilution of a rabbit anti-PF4 polyclonal antibody (ab9561; Abcam, Cambridge, UK) in 5% BSA for 16 hrs. The membranes were washed 3 times with 1xTBS containing 0.05% Tween and incubated with a 1:10000 dilution of HRP-linked goat anti-rabbit IgG polyclonal antibody (Cell signaling Technology, Danvers, MA, USA). The expression of integrin α2b protein was detected by using a 1:1000 dilution of a rabbit integrin αIIb monoclonal antibody (D8V7H; Cell signaling Technology, Danvers, MA, USA) for 16 hrs followed by incubation with a 1:5000 dilution of HRP-linked goat anti-rabbit IgG polyclonal antibody (#7074 s; Cell signaling Technology, Danvers, MA, USA) for 1 hr. The protein levels were normalized against expression of GAPDH protein. Levels of GAPDH protein were determined using a 1:5000 dilution of a mouse anti-GAPDH monoclonal antibody (sc-32233; Santa Cruz Biotechnology Inc, Texas, USA) followed by a 1:10000 dilution of a HRP-linked rabbit anti-mouse IgG polyclonal antibody (Pierce, Rockford, IL, USA). All signals were visualized signal by adding the chemiluminescent ECL substrate (Boster Biological Technology, CA, USA) and exposure to X-ray film.

### Statistical analysis

Statistical analysis of data was performed using an unpaired *t* test. Data are reported as mean ± standard deviation (SD) and the graphs were plotted using GraphPad Prism version 5.0. Spearman correlation coefficient (rs) was calculated to determine the correlation between platelet activation and prothrombin fragment 1 + 2. A *p*-value less than 0.05 was considered statistically significant.

## Supplementary information

supplementary figure
