# Supplementary material for: Platelet proteome reveals specific proteins associated with platelet activation and the hypercoagulable state in β-thalassmia/HbE patients
Source: Sci Rep. 2019 Apr 15;9:6059. doi: 10.1038/s41598-019-42432-2 (PMC6465338; doi:10.1038/s41598-019-42432-2)

Supplementary information

Platelet proteome reveals specific proteins associated with platelet activation and the hypercoagulable state in β-thalassmia/HbE patients

Puangpaka Chanpeng 1, Saovaros Svasti 2, Kittiphong Paiboonsukwong 2, Duncan R. Smith3 and Kamonlak Leecharoenkiat 1,*

4 replicated 2D gels of healthy volunteers

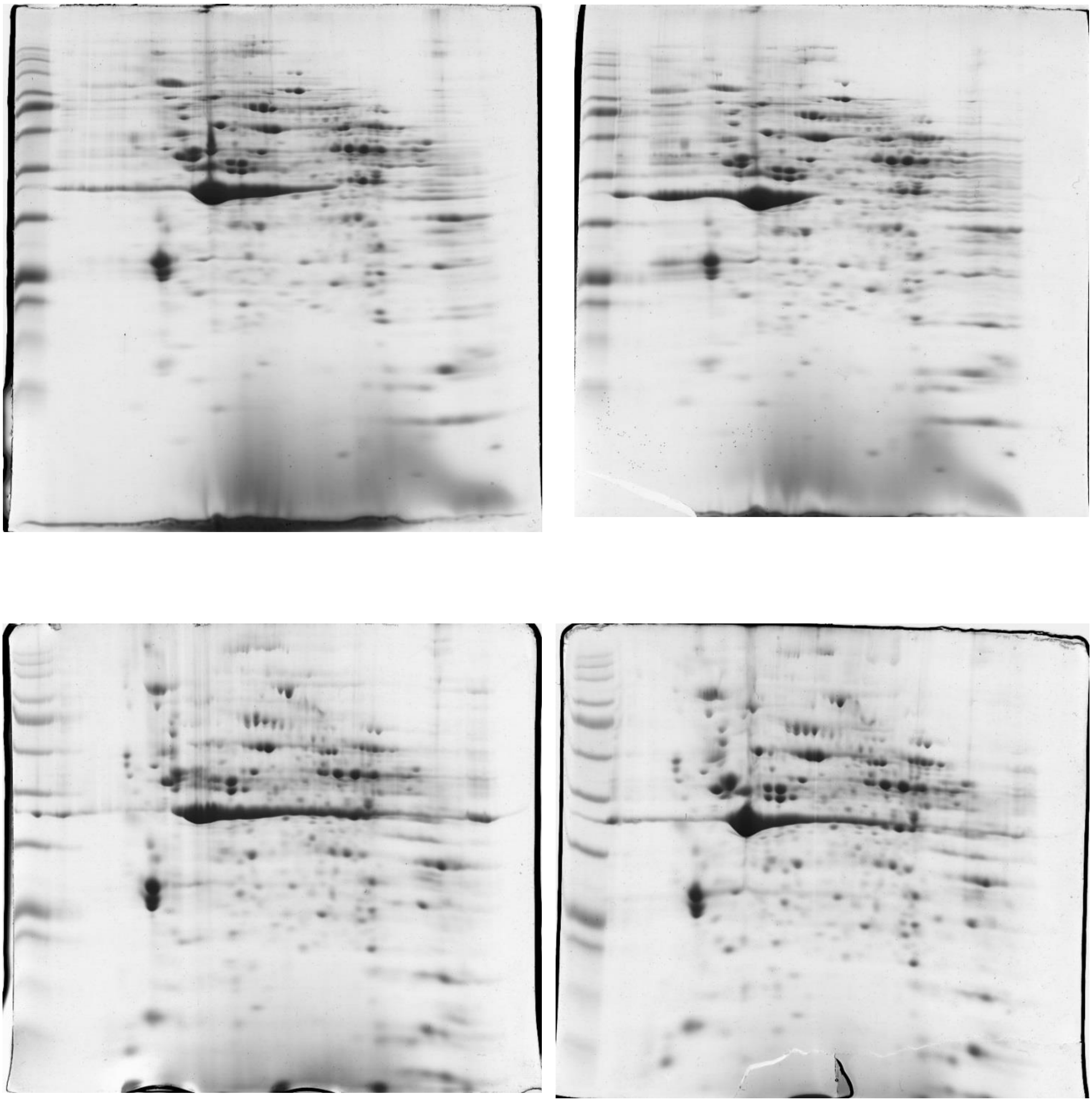

4 replicated 2D gels of non-splenectomized β- thalassemia/HbE

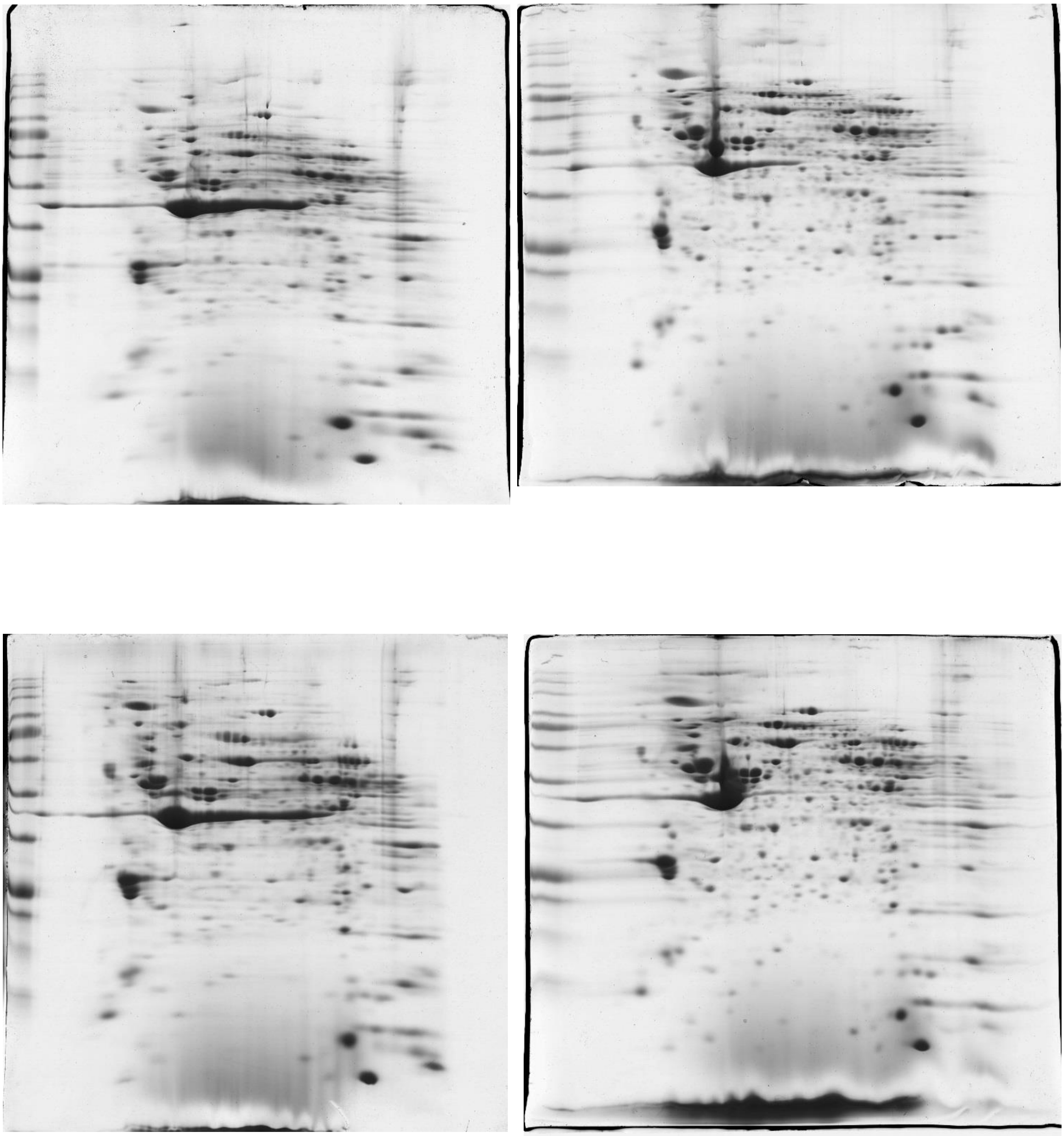

Western blot analysis of PF4 normalized against GAPDH in healthy volunteers compared to the β- thalassemia/HbE patients

PF4

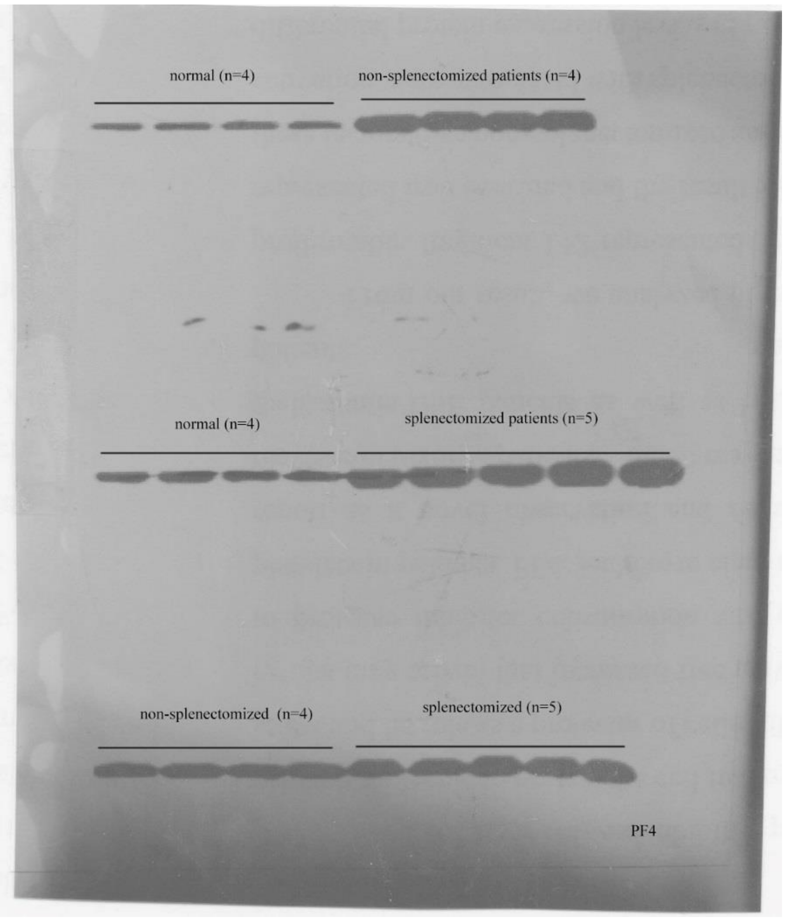

GAPDH for normalized PF4

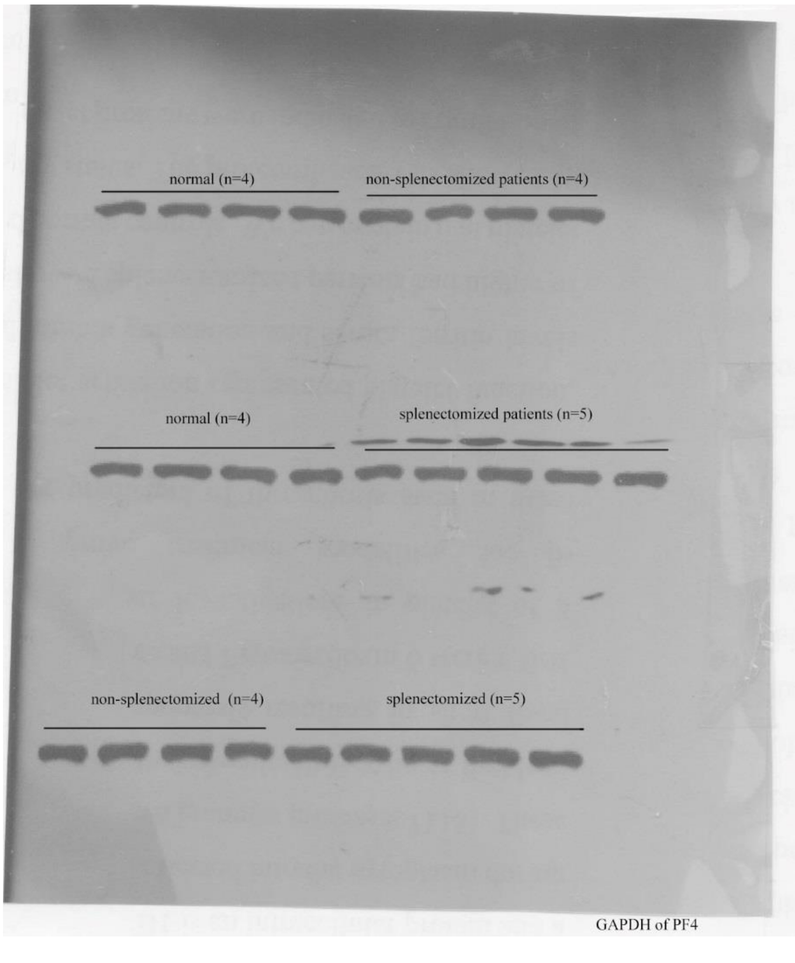

Western blot analysis of Integrin αIIb normalized against GAPDH in healthy volunteers compared to the β- thalassemia/HbE patients

Integrin αIIb

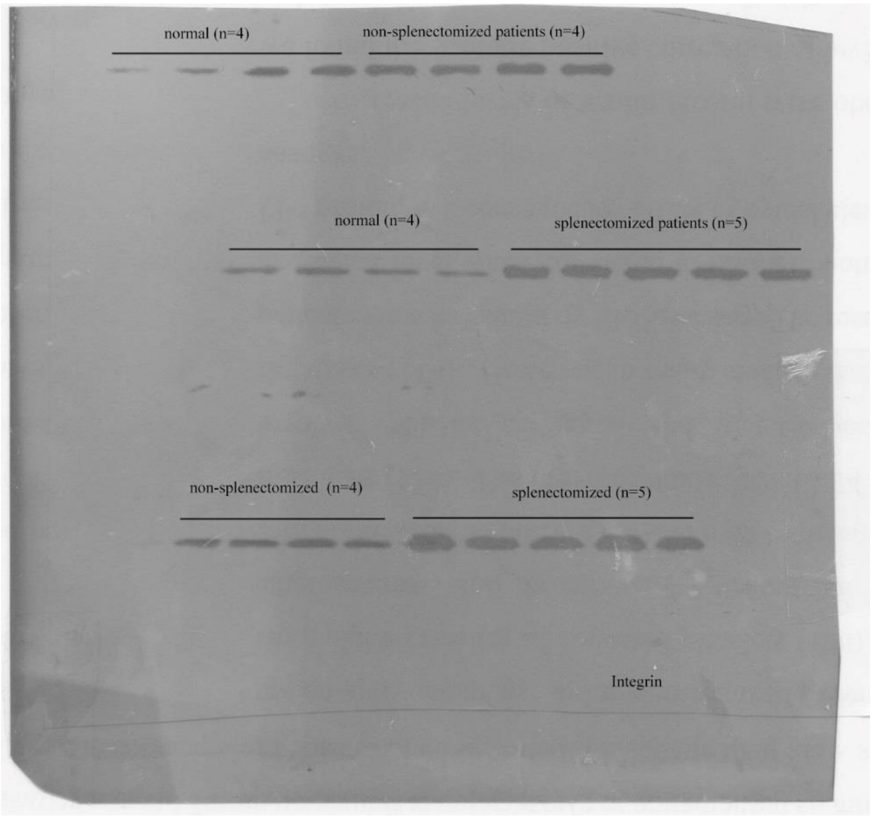

GAPDH for normalized Integrin αIIb

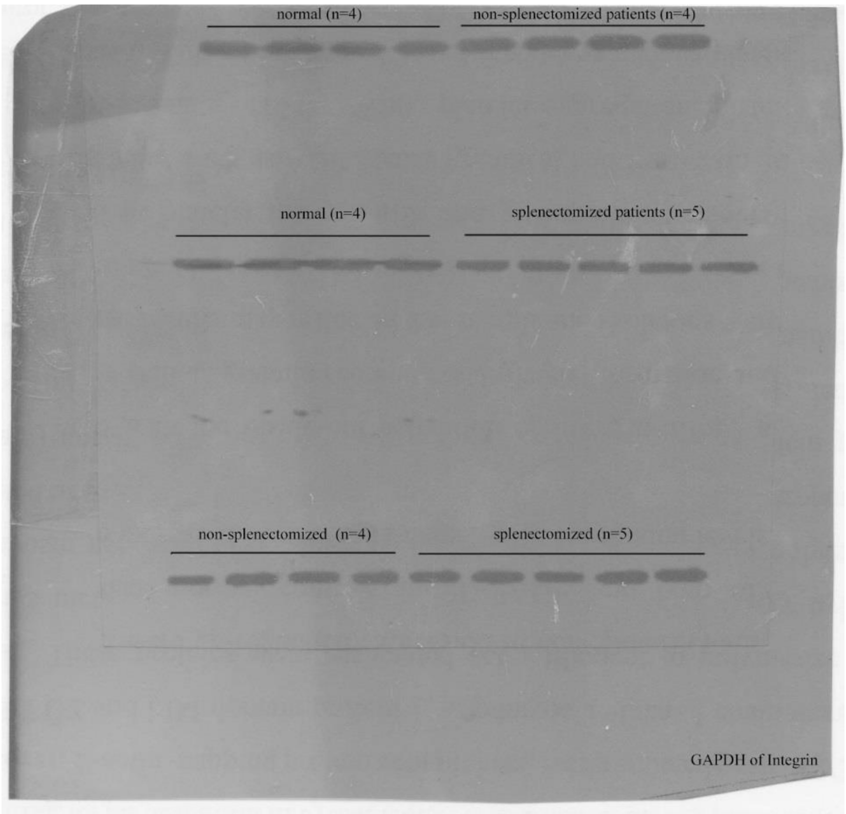

Supplement: Supplementary file 1 — supplementary figure [file 41598_2019_42432_MOESM1_ESM.pdf]
